# Topical vitamin A treatment of recalcitrant common warts

**DOI:** 10.1186/1743-422X-9-21

**Published:** 2012-01-17

**Authors:** Anca Gaston, Robert F Garry

**Affiliations:** 1 Faculty of Health Sciences, School of Kinesiology, The University of Western Ontario, London, ON N6A 3 K7, Canada; 2 Department of Microbiology and Immunology, Tulane University, 1430 Tulane Avenue, New Orleans, LA 70112, USA

## Abstract

**Background:**

Common warts (*verruca vulgaris*) are benign epithelial proliferations associated with human papillomavirus (HPV) infection. Salicylic acid and cryotherapy are the most frequent treatments for common warts, but can be painful and cause scarring, and have high failure and recrudescence rates. Topical vitamin A has been shown to be a successful treatment of common warts in prior informal studies.

**Case:**

The subject is a healthy, physically-active 30 old female with a 9 year history of common warts on the back of the right hand. The warts resisted treatment with salicylic acid, apple cider vinegar and an over-the-counter blend of essential oils marketed for the treatment of warts. Daily topical application of natural vitamin A derived from fish liver oil (25,000 IU) led to replacement of all the warts with normal skin. Most of the smaller warts had been replaced by 70 days. A large wart on the middle knuckle required 6 months of vitamin A treatment to resolve completely.

**Conclusion:**

Retinoids should be further investigated in controlled studies to determine their effectiveness in treating common warts and the broad range of other benign and cancerous lesions induced by HPVs.

## Background

Human papillomaviruses (HPVs) are the causative agents of a variety of benign and cancerous lesions of the skin and other epithelial surfaces. At least 189 HPV genotypes have been described [1]. Most HPV types are associated with one or a few histopathologically distinct types of lesions and may be restricted to a particular location on the body. HPV types 2, 4, 26, 29 and others are responsible for common warts (*verruca vulgaris*), which are slightly raised rough surface epithelial proliferations that occur most often on the hands, but can also grow elsewhere on the body. Other types of warts include plantars warts (*verruca plantaris*) that occur most commonly on the soles of the feet (HPV 1 and others) [2], flat warts (*verruca plana*) usually appearing on the face (HPV 3, 10, 38 and others) [3], butcher's warts of the hands and fingers (HPV 7) [4], and oral, gentialoranogenital warts (*condyloma acuminata*; HPV 6, 11, 16, 18 and many others) [5]. While common warts can affect patients' quality of life by causing adverse psychological effects or negative social perception, certain types of HPV may induce life-threatening malignancies. HPV is associated with virtually 100% of cervical cancers, and a high portion of cancers of the penis and anus [5-7]. HPV 16 has also been strongly associated with various head and neck cancers, including head and neck squamous cell carcinoma and oropharyngeal carcinoma of the tonsils [8-10]. The incidence of HPV-induced oral cancers appears to be increasing [11].

Many different treatments have been described for HPV-induced lesions. Salicylic acid (SCA) and cryotherapy, which are intended to kill HPV-infected cells, are the most frequently employed treatments for common warts by dermatologists. Over-the-counter (OTC) versions of these treatments are also available. Numerous studies, albeit with highly variable protocols, have examined the efficacy of SCA and cryotherapy for treating HPV-induced lesions (reviewed in [12]). The efficacy of these treatments is low, and there is a high rate of recrudescence and adverse effects (such as scarring). Although well-controlled clinical trials have not been performed, retinoids are promising alternative treatments for warts [13-17]. Topical vitamin A was an effective treatment of common warts in a prior informal study [18]. Here, we present a well-documented case of topical vitamin A treatment of recalcitrant common warts.

## Case presentation

**Figure 1:**
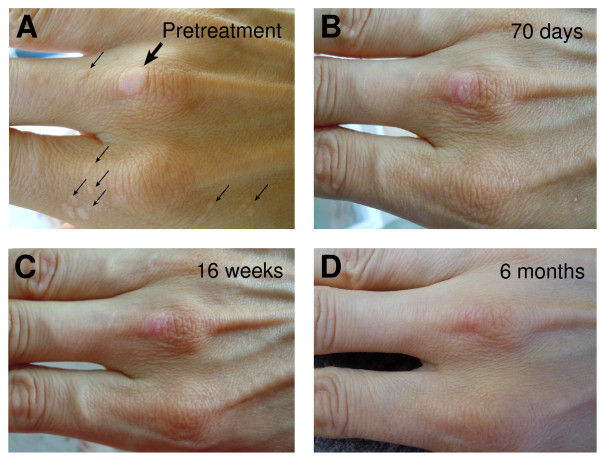
**Progressive clearance of *verrucae vulgaris *following daily topical application of vitamin A**. Appearance of warts prior to vitamin A treatment (Panel **A**) and at 70 days (Panel **B**), 16 weeks (Panel **C**) and 6 months (Panel **D**). Large arrow indicates large wart on knuckle requiring the longest time to resolve. Small arrows indicate smaller warts cleared by 16 weeks.

The subject is a healthy 30 year old woman of European descent with an uneventful medical history. There was a large (approximately 9 mm in diameter; large arrow Figure 1A) common wart on the middle knuckle of the right hand as well as 23 smaller warts (1 mm to 4 mm) at the base of and behind the knuckle of the index finger (small arrows, Figure 1A). The large wart was first noticed 9 years earlier. Within 1-2 years it had reached its maximum size as well as spread to multiple surrounding sites. Because of her occupation as a piano teacher, the subject reported experiencing considerable psychological distress over the visibility of the warts and the need to frequently use her hands in front of others. Over the course of the previous 2 years, several treatments for common warts on the right hand were unsuccessful despite perfect compliance. The treatments attempted included two courses of essential oil blends which were applied to all warts three times daily as directed. The initial preparation was marketed OTC for wart treatment, guaranteed by the manufacturer to be effective within 8 weeks, and consisted of cedrus, lycea, malaleuca alternafolia, and limonium. After 3 months of perfect application resulted in no change, a second specially-formulated stronger blend was applied three times daily for an additional 2 months. This consisted of a stronger concentration of the same aforementioned essential oils plus juniper and cypress. It resulted in no change. The second unsuccessful treatment was an OTC 40% salicylic acid preparation that was applied only to the large wart for 3 months of daily application. The salicylic acid treatment resulted in very little benefit and a plateau effect after 3 weeks with no further improvement. A third unsuccessful treatment was apple cider vinegar applied to two of the smaller warts. The supradermal portion of the warts sluffed off after a few days of apple cider vinegar treatment, but promptly regrew. In addition, this treatment resulted in moderate to severe irritation of the surrounding skin.

Throughout the course of the failed attempts at treatment, the number of warts increased. All warts had returned to their original appearance prior to treatment with vitamin A. The source of vitamin A was natural fish liver oil (NOW^® ^Foods, 25,000 IU softgels, Manufacturer SKU: 733739003409). A needle was used to puncture the softgels and the oil was applied topically to the warts every night prior to sleep. The oil was allowed to soak into the tissue before the excess oil was removed with a dry towel. Soap was not used to wash the hands until the morning. On 3-4 days of the week, the oil was applied a second time, usually around midday. The daily topical application of vitamin A led to replacement of all the warts with normal skin. Most of the smaller warts had been replaced with normal skin by 70 days (Figure 1B). The largest wart on the middle knuckle of the right hand, although replaced by mostly normal tissue after 4 months of treatment (Figure 1C), required 6 months of vitamin A treatment to completely resolve (Figure 1D). The slight discoloration of the tissue likely represents residual scar tissue from prior unsuccessful treatments. Interestingly, during the course of treatment three tiny warts on the left hand also disappeared even though vitamin A was not applied to them.

## Conclusions

The case reported here is similar to experiences in prior informal studies suggesting that topical vitamin A is an effective treatment for *verruca vulgaris*. Topical vitamin A should be further investigated as an alternative to the most frequently-utilized wart treatments, cryotherapy and SCA, which have relatively low cure rates [19-21]. In contrast to destructive or dermonecrotic treatments, topical vitamin A treatment for warts is not painful and does not lead to scarring. On the other hand, the treatment may require several months for complete resolution of the lesions, which can affect compliance. As in the current case, warts with scar tissue as a result of prior tissue destroying treatments, such as SCA, may require longer vitamin A treatment. The location and size of the wart may also affect time to resolution. Large plantar warts on the feet typically take several months to resolve with topical vitamin A. Treatments such as SCA and cryotherapy have a high rate of recrudescence that is not seen with topical vitamin A treatment. Destructive treatments that do not affect HPV replication must eliminate all virus from the deep tissue, or commonly the wart will reestablish quickly.

There are a number of plausible mechanisms by which retinoids may affect HPV-induced lesions. Warts display abnormal keratin expression [22-24]. Retinoids, such as vitamin A, regulate epithelial cell differentiation and keratin expression [25]. HPV cannot complete its replication cycle in cultured cells suggesting that differentiation of epithelia cells in tissues is important for HPV production [26]. HPV replication appears to be synchronized with epithelial cell differentiation [27,28]. Vitamin A may disrupt the interplay of HPV replication and epithelial cell differentiation, thereby allowing normal tissue to replace the warts. Previous studies have suggested that retinoids may also affect HPV transcription or replication [29,30]. Furthermore, HPV infection may alter retinoid signaling [31,32]. Immune mechanisms may also be involved in wart clearance [33]. Vitamin A treatment may increase or prolong expression of HPV T or B cell antigens allowing clearance of the warts by immune mechanisms. The observation that three small warts on the left hand of the current subject resolved simultaneously even though vitamin A was not applied directly to them, suggests the possibility that vitamin A may evoke or potentiate immune responses to warts. While it is unlikely that topical application could increase the systemic levels of vitamin A substantially (hypervitaminosis A), a modest systemic elevation of vitamin A levels by the topical treatment may have affected the small warts on the left hand.

Furthermore, warts can have psychologically devastating effects on patients' lives [34] and the emotional impact of any treatment should not be overlooked. Treatment with SCA, for example, requires carefully coating the wart with a highly noticeable white film and may not be viable for sufferers who are already self-conscious or whose warts are on their face. In contrast, vitamin A oil is colourless, easy to apply, and quickly absorbed into the tissues. In addition, it does not irritate surrounding skin or lead to drying or flaking. Because vitamin A causes the wart to slowly dissolve and disappear, it represents an inconspicuous treatment that does not attract further attention to the wart and is unlikely to cause additional psychological distress.

Because warts may "spontaneously" resolve, it is possible that clearance of the warts after treatment with vitamin A in this case was due to chance timing. However, this is unlikely given that the warts had only increased in size and number over the preceding 9 years. In addition, the case is typical of subjects in a prior informal series in which warts always began to clear within a few weeks of initiating topical vitamin A treatment [18]. Tretinoin (Retin A) and other retinoids besides vitamin A have been successfully used in limited studies for the treatment of warts [13-17]. The current case adds to the literature suggesting that retinoids should be further investigated in controlled studies to determine their effectiveness in treating common warts and the broad range of other benign and cancerous lesions induced by HPVs.

## Consent

Informed consent was obtained from the subject for publication of this Case Report and accompanying images.

## Abbreviations

HPV: Human papillomavirus; OTC: Over-the-counter

## Competing interests

The authors declare that they have no competing interests.

## Authors' contributions

AG performed the investigations described in this study. RFG participated in its design. AG and RFG wrote and edited the manuscript. Both authors read and approved the final manuscript.
